# Inter-Species Cross-Seeding: Stability and Assembly of Rat - Human Amylin Aggregates

**DOI:** 10.1371/journal.pone.0097051

**Published:** 2014-05-08

**Authors:** Workalemahu M. Berhanu, Ulrich H. E. Hansmann

**Affiliations:** Department of Chemistry and Biochemistry, University of Oklahoma, Norman, Oklahoma, United States of America; University of Maryland School of Medicine, United States of America

## Abstract

Diseases such as type 2 diabetes, Alzheimer’s and Parkinson’s share as common feature the accumulation of mis-folded disease-specific protein aggregates into fibrillar structures, or plaques. These fibrils may either be toxic by themselves, or act as reservoirs for smaller cytotoxic oligomers. This suggests to investigate molecules as potential therapeutics that either reduce fibril formation or increase fibril stability. One example is rat amylin, which can inhibit aggregation of human amylin, a hallmark of type 2 diabetes. In the present paper, we use molecular dynamics to compare the stability of various preformed aggregates, built out of either human amylin, rat amylin, or mixtures of both. We considered two types of fibril-like oligomers: a single-layer in-register conformation, and a double-layer conformation in which the first U-shaped layer consists of rat amylin and the second layer of human amylin. Our results explain the weak amyloid-inhibiting properties of rat amylin and suggest that membrane leakage due to pore formation is responsible for the toxicity of rat amylin observed in a recent experiment. Together, our results put in question the use of rat amylin or the similar FDA approved drug pramlintide as an inhibitor of human amylin aggregation. They also point to mixed human-rat amylin fibril-like oligomers as possible model-systems for studies of amyloid formation that involve cross-species transmission.

## Introduction

In human amyloid diseases, protein mis-folding triggers the formation of amyloid oligomers and fibers that can cause cell death leading to either localized or systemic organ failure [1]. One example is human amylin whose main physiological function is suppression of food intake and inhibition of gastric contractions [2]. Human amylin is one of the most amyloidogenic proteins [3]. It’s aggregates damage not only β-cells, leading to the reduction of insulin secretion [4], [5], [6] in type 2 diabetes, but also cells in other organs including kidneys [7], heart [8] and the cerebrovascular system [9]. Likely, the main toxic species are not mature fibers but amyloid oligomers [10], [11], with the fibrils potentially acting as reservoirs for the toxic oligomers. This suggests as potential therapeutics molecules that stabilize fibers and therefore shift the equilibrium from smaller, toxic entities towards the fibrillar state [12], [13]. A candidate for such molecules is rat amylin, which due to its high sequence similarity [14] binds strongly to human amylin, but is not amyloidogenic under physiological conditions [15] (and rats therefore do not develop type 2 diabetes [16], [17]). Mixing equal molar concentrations of rat with human amylin leads to a deposition of the non-aggregating rat amylin onto human amylin fibrils resulting in a weak aggregation inhibitor activity [18].

However, the interaction mechanisms that stabilizes these mixed amyloid fibrils are not known, as their structures are difficult to characterize. In the present study, we use multiple long-time molecular dynamics simulations [19], [20], [21] to probe the mechanism by which the non-aggregating rat amylin can grow on the surface of human amylin. For this purpose, we investigate the contribution of specific β-strand to β-stand and β-sheet to β-sheet interactions on the elongation and lateral growth of single and double layer models (with both C-terminal–C-terminal and N-terminal–N-terminal interfaces) of human amylin, rat amylin and mixed rat-amylin oligomers. Our aim is to probe what types of intermolecular interactions reduce the cross species barrier and encourage cross-seeding of human and rat amylin fibril-like oligomers. Such molecular insight may not only help with the rational design of components that improve upon rat amylin’s inhibitory effects on human amylin aggregation, but also lead to a better understanding of the mechanism of cross-seeding in amyloid diseases that are caused by cross-species transmission.

## Methods

### Structural Models Details

Both human and rat amylin are built out of 37 residues, of which the first 17 residues (the N-terminal region) are identical in both species, including the two positively charged residues, K_1_ and R_11_. The most prominent difference in sequence is the presence of three prolines (which are known to break β-strands) in the C-terminus of rat amylin, at positions 25, 28 and 29 [16]. At position 23, phenylalanine, an aromatic residue, is replaced in rat amylin with the aliphatic leucine. The histidine at position 18 in human amylin is replaced in rat amylin by another basic residue, arginine; and the aliphatic isoleucine at position 26 by valine, which is also aliphatic.

As of today, no one has crystallized full-length human amylin. Amyloid fibrils exhibit polymorphism due to differences in the packing at the interface between the two proto-filaments. This polymorphism is also reflected by the variety of fibril models of amylin [27]. Early models are made out of three β-strands in a monomer [22], [23], but the most recent high-resolution amylin fibril structures are U-shaped and formed by only two β-strands. Examples are the models proposed by Wiltzius et al. [24], Luca et al. [25], and Bedrood et al. [26]. The X-ray derived models differ only slightly in the details of side-chain packing and have been shown to be more stable than the NMR Tycko model [28], [29], [30], [31]. For instance, previous molecular dynamics simulations indicate that these X-ray models [28], [29], [30] have more closely interlocked side chains of the β-strands that tighten the binding of two β-sheets making them more compact and stable than the solid state NMR model proposed by the Tycko group. The topology of these X-ray models is similar to that reported by Luca et al [25]) which is based on solid state NMR. Note that the U-shaped human amylin structure is similar to recent fibril models determined from brain tissue of patients. We believe that this lends support for the X-ray model as the most likely candidate structure in investigations of the mechanism which stabilizes the fibers [32]. For these reasons, we use it as start structure [24] in our study.

The full-length X-ray human amylin fibril model has a characteristic U-shaped β-strand-loop-β-strand motif and is formed from the atomic structure of segments 21−27 and 28−33 taking into account supporting biochemical and structural data. The X-ray model shows a tighter side chains inter-digitation than those deduced by ssNMR [23], and EPR [26], where the two strands are made of residues 8−17 and 28−37, with the loop region located at residues 18−27. We have downloaded this human amylin fibril model from the web-site http://people.mbi.ucla.edu/sawaya/jmol/fibrilmodels/. A key assumption in our study is that rat amylin adopts a single-layer U-shaped structure that is similar to the one observed in human amylin. This is why we use the X-ray derived β-strand–turn−β-strand motif fibril model of human amylin as a building block to construct rat amylin by changing the six differing residues to those of the rat sequence (i.e. H_18_R, F_23_L, A_25_P, I_26_V, S_28_P and S_29_P), keeping their side chain orientation and backbone conformation. We believe that the existing experimental evidence supports our assumption. For instance, a recent study on lyophilized rat amylin, dissolved in 20 mM Tris-HCl, indicates that rat amylin forms fibrils, which bind to Congo red and therefore are structurally similar to other amyloids. It has also been shown that rat amylin peptides can form its own amyloid β-sheet when provided with a human amylin β-sheet as template; and that such cross seeding between peptides with large degree of sequences similarity (such as human and rat amylin) requires conformational compatibility [33], [34].

**Figure 1:**
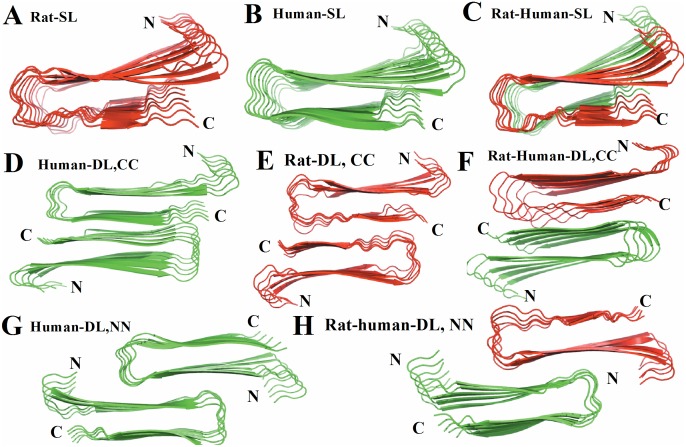
Structural details of the single and double layer decamers of rat amylin, human amylin and rat-human amylin mixtures. (A) Single layer conformation of human amylin, (B) single layer conformation of rat amylin, (C) single layer conformation of human-rat complex, (D) Double layer conformation of human amylin with CC interface (E) Double layer conformation of rat amylin with CC interface, (F) Double layer conformation of rat and human amylin mixtures with CC interface, (G) Double layer conformation of rat amylin with NN interface and (H) Double layer conformation of rat–human amylin complex with NN interface. Different colors are applied for the rat (red) and human amylin (green).

The rat and human amylin oligomers can either be combined to form a longer proto-filament (elongation, single layer), or be merged via either N or C-terminal contacts to form a proto-filament pair (thickening, double layer) [35]. We assume that the interaction between the human and rat amylin occurs through the β-strand motif [20], [19]. The single layer model is build out of a human amylin fibril-like oligomer made out of five chains followed by a rat-amylin oligomer that also consists of five chains. The double-layered model is constructed by placing the two five-stranded fibril-like oligomers in such a way that either C-terminal–C-terminal or N-terminal–N-terminal facing each other, and afterwards maximizing the overlap between the two interfaces. Previous molecular dynamics studies of the energetics and the structural stabilities of monomers and small oligomers (up to pentamers) indicate that the U-shaped form of our initial human amylin fibrillar conformation is stable in trimers, tetramers and pentamers, where the two parallel in-register β-sheets as well as the connecting turn are preserved. On the other hand, the monomer and dimer predominantly exist in conformations that differ from the larger oligomers and the fibril structure [36], [28], [37]. This suggests to use trimers, tetramers or pentamers rather than dimers and monomer as seeds for fibril formation, which is in agreement with experimental observations [38]. For these reasons, we have used in our simulation the described blocks of five chains of rat or human amylin peptides as our start structures. In all cases, the single and double layer models (**Table 1** **and** **Figure 1**) are minimized afterwards in 500 steps with the steepest decent algorithm keeping the protein backbone restrained. Note that the CC interface in the double layer models is dominated by polar interactions (N_35_–A_25_, G_33_–L_27_, N_31_–S_29_, L_27_–G_33_ and A_25_–N_35_), while the NN interface consists of a combination of polar residue of T_9_, charged residue of R_11_, and hydrophobic residues of A_13_, F_15_, and V_17_. The CC interface has a larger steric zipper than the NN interface. Additionally, rat amylin and the mixed rat-human amylin complexes contain multiple prolines, known to break β-sheets, that may influence the stability of the preordered fibrillar structures.

**Table 1:** Single layer and double layer decamer models and simulations conditions.

*System*	*#Atoms of peptide/* *#Atoms Water/Cl^−^*	*Simulation box dimensions* *(x, y, z [Å])*	*Simulation time, ns*
Rat-amylin (Rat-SL)	5350/36665/20	106.8, 106.8, 106.8	900 ns (300×3)
Human-amylin (Human-SL)	5340/36675/20	106.8, 106.8, 106.8	900 ns (300×3)
Rat-human amylin complex* (Rat-human-SL)	5340/36396/20	106.6, 106.6, 106.6	900 ns (300×3)
Rat-amylin (Rat-DL, CC)	5330/36695/20	106.8, 106.8, 106.8	900 ns (300×3)
Human-amylin (Human-DL, CC)	5345/36667/20	107.0, 107.0, 107.0	900 ns (300×3)
Rat-human amylin complex* (Rat-Human-DL, CC)	5345/36677/20	106.9, 106.9, 106.9	900 ns (300×3)
Human-amylin (Human-DL, NN)	5340/36681/20	106.9, 106.9, 106.9	900 ns (300×3)
Rat-human amylin complex* (Rat-Human-DL, NN)	5350/39644/20	108.1, 108.1, 108.1	900 ns (300×3)

SL marks single layer decamers and DL double layer decamers. The symbol * marks the mixed rat-human amylin complexes, where the first five strands are from the human amylin sequences and the last five strands are form rat amylin sequence. NN strands for N-terminal-N-terminal interface and CC strands for C-terminal-C-terminal interface.

### Details of Molecular Dynamics Simulations

Our molecular dynamics simulations utilize the AMBER ff99SB force field [39] in combination with explicit water (TIP3P) [40], [41], as implemented in GROMACS program version 4.5.5 [42]. Hydrogen atoms are added with the *pdb2gmx* module. For all proteins, we put the start configuration in the center of a cubic box, with at least 12 Å between the solute and the edge of the box. Using periodic boundary conditions we calculate electrostatic interactions by the particle-mesh Ewald (PME) algorithm [43],[44]. Hydrogen atoms are constrained with the LINCS [45] algorithm while for water the Settle algorithm is used [46]. The amino acids are ionized according to their pKa values, and chloride ions are added as needed to neutralize the system. A time step of 2 fs is used. The temperature of 310 K is kept constant by the Parrinello-Donadio-Bussi algorithm [47] (τ = 0.1 fs) which is similar to Berendsen coupling but adds a stochastic term to ensure convergence to a canonical ensemble [47],[48]. In a similar way, we keep the pressure constant at 1 bar by the Parrinello-Rahman algorithm [49] (τ = 1 fs). After minimizing the energy of the solvated start configuration by steepest descent, followed by conjugate gradient, the system is equilibrated in two steps of 500 ps, first in an NVT ensemble and afterwards in an NPT ensemble at 1 bar. After reaching equilibrium, each system is followed over 300 ns to monitor how the oligomer structures evolve with time, with the data saved at 4.0 ps intervals for further analysis. For each system (**Table 1**), we run three distinct simulations with different initial velocity distributions. This allows us to test that we reached equilibrium and guarantees three independent sets of measurements.

The resulting molecular dynamics trajectories are analyzed with the tool set of the GROMACS package. Specifically, we monitor conformational changes and the stability of the oligomer models through the time evolution of root means square deviations of the Cα atoms (RMSD), root-mean-square fluctuation (RMSF), hydrophobic contact distances and hydrogen bonds. The quantities are measured with the *g_hbond* and *g_dist* modules in GROMACS. Hydrogen bonds are defined by a distance cut-off between donor and acceptor of 0.36 nm and an angle cut-off of 30°. The DDSP program is used to analyze secondary structure [15]. Configurations are visualized using PyMOL [50].

## Results and Discussion

We test structural stability and characteristics of the various models by calculating the root mean square deviation (RMSD) of backbone atoms, root mean square fluctuations (RMSF), secondary structure, number of hydrogen bonds, the inter-sheet distances; by visual structural analysis, and by monitoring water permeation across the single layer and double layer systems. We first present our results on the human amylin, followed by rat and finally the mixed human-rat amylin fibril models.

### Human Amylin

**Figure 2:**
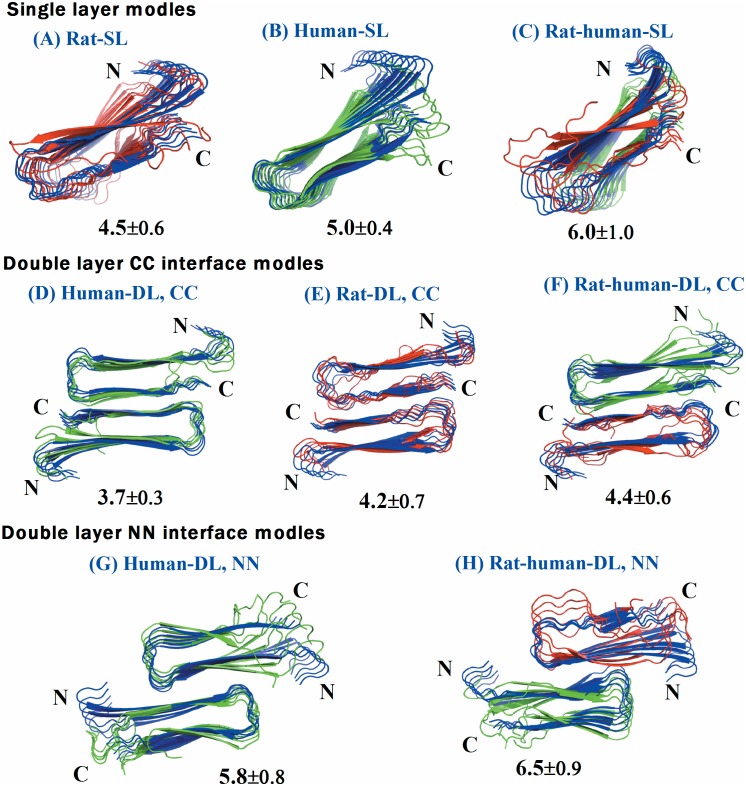
The structural changes in each model, from the trajectory with the largest average root-mean-square-deviations, at the end of 300 ns of molecular dynamics in explicit solvent (water molecules omitted for clarity). A) Single layer conformation of human amylin, (B) single layer conformation of rat amylin, (C) single layer conformation of human-rat complex, (D) Double layer conformation of human amylin with CC interface (E) Double layer conformation of rat amylin with CC interface, (F) Double layer conformation of rat and human amylin mixtures with CC interface, (G) Double layer conformation of rat amylin with NN interface and (H) Double layer conformation of rat–human amylin complex with NN interface. The segments that are colored yellow are the N terminal segments (residue 8–17) and the C terminal segment (residue 28–37). Different colors are applied for the rat (red) and human amylin (green). The initial structures are depicted in blue. Root-mean-square-deviation values calculated for each peptide with respect to the start configurations are included in parentheses.

**Figure 3:**
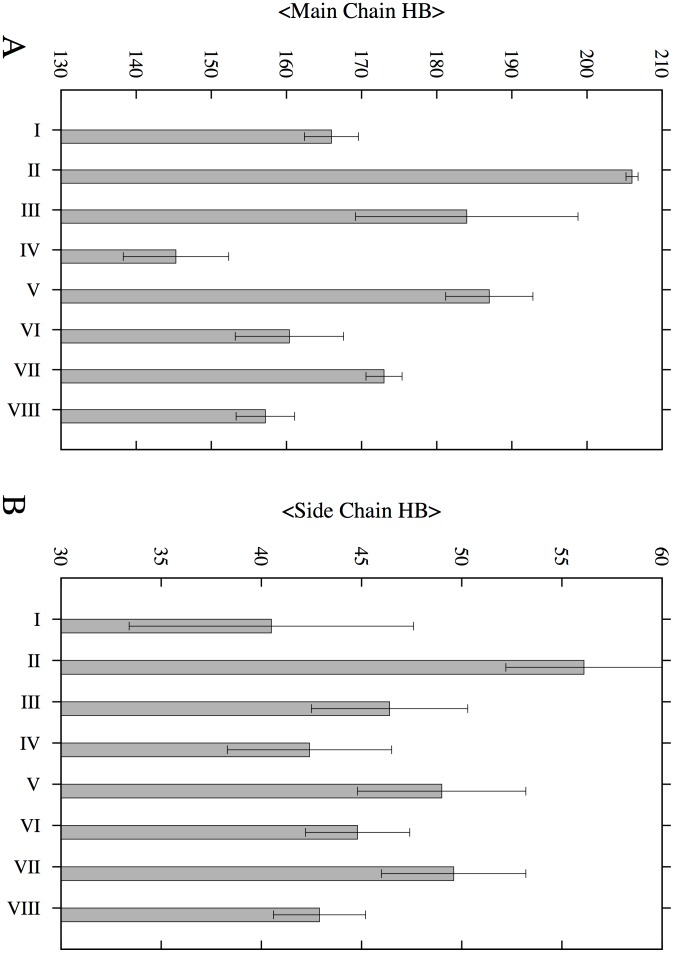
Average number of main chain and side chain hydrogen bonds. (A) Total number of main chain hydrogen bonds; (B) total number of side chain hydrogen bonds. Legend: (I) Single layer conformation of rat amylin, (II) single layer conformation of human amylin, (III) single layer conformation of human-rat complex, (IV) Double layer conformation of rat amylin with CC interface (V) Double layer conformation of human amylin with CC interface, (VI) Double layer conformation of rat and human amylin mixtures with CC interface, (VII) Double layer conformation of human amylin with NN interface and (VIII) Double layer conformation of rat–human amylin complex with NN interface.

Visual inspection of the initial and final structures for human amylin SL (single layer) and DL (double layer) models shows that the U-shape of the human amylin conformation is fully preserved (**Figure 2** and **Figure S1**). Fibrils such as the ones studied here are stabilized in part by a large number of hydrogen bonds including such between each strand and its neighbors [51], [19], [52], [53]. Hence, the gain or loss of hydrogen bonds quantifies the changes in structural stability of our fibrillar constructs. Counting main-chain and side-chain hydrogen bonds and averaging them over all three trajectories (**Figure 3**), we find that hydrogen bonding is more important for the single layer human amylin, which has a larger number of hydrogen bonds, than the double layer models where more stands are exposed to the solvent (four strands versus two strands in the single layer model). The inter-peptide hydrogen bonding in both double layer models follows a similar trend during the simulation, with a slightly larger increase of main chain hydrogen bonds for the model with CC interface than seen in the NN interface double layer model. The number of the side chain hydrogen bonds was similar in both models. The NN interface double layer human amylin is stabilized due to face-to-face contact between the hydrophobic amino acid F and V side chains (**see** **Table 2**) and retains the double layer β-hairpin. This model has a slightly larger average root-mean-square deviation (**Figure 2** and **Figure S1**) than the experimentally observed double-layer model with CC interface [24], [25]. This is because in the model with CC interface the packing of adjacent β-sheet layers is tighter than in the model with NN interface (**Table 3**), increasing the stability of the CC model: the average root-mean-square deviation between start and final configuration is about 3.5 Å. This suggests that the β-strand motif of the C terminus with its larger interface (compared to the NN interface) serves as an anchor between the two hairpin units in the double layer, limiting their flexibility, and as a result enhances the stability of the double layer. For this reason, one finds in the experimental fibril models exclusively CC interfaces [25], [24]. Thus, the C-terminus is not only important for formation of human amylin oligomers, but it also stabilizes the fibril via its packing interactions, and has to be buried within the fibril. Compared to the C-terminus, the N-terminus is more flexible, but the distances measured in our simulations of human amylin double layer models with NN interface are within the range of experimentally observed inter-sheet distances. Hence, our simulations suggest that NN interface packing could be a possible source for polymorphism [54], [55], [56] (see **Table 2** and **Figure 2**). However, due to the smaller size of the steric zipper and since burring the charged residues R_11_ [57] is difficult, such forms may not be as stable as aggregates bound through their CC interface.

**Table 2:** Face to face contact distances of NN interface double layers of human amylin and cross-seeded oligomers of human amylin|rat amylin.

<F_15_/V_17_>	Human amylin oligomer			<F_15_/V_17_>	Human-rat amylin complex		
	Sh_1_−St_2_/Sh_2_−St_2_*	Sh_1_−St_3_/Sh_2_−St_3_	Sh_1_−St_4_/Sh_2_−St_4_		Sh_1_−St_2_/Sh_2_−St_2_	Sh_1_−St_3_/Sh_2_−St_3_	Sh_1_−St_4_/Sh_2_−St_4_
Run1	8.5 (0.4)	8.4 (0.5)	9.0 (0.5)	**Run1**	10.0 (0.3)	9.8 (0.4)	9.7 (0.6)
Run2	8.5 (0.4)	8.4 (0.5)	9.0 (0.7)	**Run2**	9.2 (0.4)	8.9 (0.5)	8.4 (0.7)
Run3	8.4 (0.5)	8.7 (0.5)	9.0 (0.6)	**Run3**	8.8 (0.4)	8.6 (0.4)	8.2 (0.5)
Mean±SD	**8.5±0.1**	**8.5±0.2**	**9.0±0.0**		**9.3±0.6**	**9.1±0.6**	**8.8±0.8**
**<V_17_/F_15_>**	Sh_1_−St_2_/Sh_2_−St_2_*	**Sh_1_−St_3_/Sh_2_−St_3_**	**Sh_1_−St_4_/Sh_2_−St_4_**	**<V_17_/F_15_>**	**Sh_1_−St_2_/Sh_2_−St_2_**	**Sh_1_−St_3_/Sh_2_−St_3_**	**Sh_1_−St_4_/Sh_2_−St_4_**
Run1	8.7 (0.5)	9.0 (0.5)	9.9 (0.5)	**Run1**	10.3 (0.3)	10.3 (0.4)	10.5 (0.6)
Run2	10.0 (0.5)	9.9 (0.5)	9.8 (0.4)	**Run2**	10.5 (0.6)	11.1 (0.6)	11.1 (0.7)
Run3	9.2 (0.8)	9.1 (0.7)	9.2 (0.8)	**Run3**	11.6 (0.4)	11.7 (0.4)	10.1 (0.4)
Mean±SD	**9.3±0.6**	**9.3±0.5**	**9.6±0.4**		**10.8±0.7**	**11.0±0.7**	**10.6±1.3**

Hydrophobic contact of C_α_−C_α_ distances (Å) between the residues F_15_/V_17_, and V_17_/F_15_ of human amylin and human-rat amylin and their hetero-assembly. *Sh = sheet and St = strand. Values are shown after excluding the first and the last chain of the β-hairpin structures.

**Table 3:** Face to face contact distances of CC interface double layers of human amylin, rat amylin and their cross-seeded oligomers (human amylin|rat amylin).

<L_27_/G_33_>	Human amylin oligomer			L_27_/G_33_	Rat amylin oligomer			L_27_/G_33_	Mixed human-rat amylin oligomer		
	Sh_1_−St_2_/Sh_2_−St_2_*	Sh_1_−St_3_/Sh_2_−St_3_	Sh_1_−St_4_/Sh_2_−St_4_		Sh_1_−St_2_/Sh_2_−St_2_	Sh_1_−St_3_/Sh_2_−St_3_	Sh_1_−St_4_/Sh_2_−St_4_		Sh_1_−St_2_/Sh_2_−St_2_	Sh_1_−St_3_/Sh_2_−St_3_	Sh_1_−St_4_/Sh_2_−St_4_
Run1	7.3 (0.3)	7.3 (0.2)	7.4 (0.3)	**Run1**	9.7 (0.6)	10.0 (0.7)	10.9 (1.1)	**Run1**	8.7 (1.6)	8.2 (0.8)	7.6 (0.5)
Run2	7.2 (0.2)	7.4 (0.2)	7.3 (0.2)	**Run2**	9.6 (0.4)	9.4 (0.5)	10.3 (0.8)	**Run2**	10.2 (0.9)	10.1 (0.7)	9.7 (0.7)
Run3	7.1 (0.4)	7.2 (0.3)	7.3 (0.3)	**Run3**	9.8 (1.5)	9.8 (1.7)	10.5 (2.1)	**Run3**	10.3 (0.6)	8.7 (0.5)	9.7 (0.4)
Mean±SD	**7.2±0.1**	**7.3±0.1**	**7.3±0.1**		**9.7±0.1**	**9.7±0.3**	**10.6±0.3**		**9.8±0.9**	**9.0±1.0**	**9.0±1.2**
**<S_29_/N_31_>**	Sh_1_−St_2_/Sh_2_−St_2_*	**Sh_1_−St_3_/Sh_2_−St_3_**	**Sh_1_−St_4_/Sh_2_−St_4_**	**P_29_/N_31_**	**Sh_1_−St_2_/Sh_2_−St_2_**	**Sh_1_−St_3_/Sh_2_−St_3_**	**Sh_1_−St_4_/Sh_2_−St_4_**		**Sh_1_−St_2_/Sh_2_−St_2_**	**Sh_1_−St_3_/Sh_2_−St_3_**	**Sh_1_−St_4_/Sh_2_−St_4_**
Run1	5.5 (0.2)	5.5 (0.2)	5.6 (0.3)	**Run1**	8.0 (0.7)	9.2 (0.7)	9.3 (1.2)	**Run1**	7.8 (0.5)	7.5 (0.3)	7.3 (0.3)
Run2	5.4 (0.2)	5.6 (0.2)	5.9 (0.3)	**Run2**	7.7 (0.4)	8.6 (0.4)	8.2 (0.4)	**Run2**	7.7 (0.4)	7.1 (0.4)	6.8 (0.5)
Run3	5.4 (0.2)	5.4 (0.2)	5.4 (0.2)	**Run3**	8.7 (0.9)	10.2 (1.1)	10.6 (1.5)	**Run3**	8.2 (0.3)	7.7 (0.3)	8.0 (0.4)
Mean±SD	**5.4±0.05**	**5.5±0.1**	**5.7±0.2**		**8.2±0.5**	**9.3±0.7**	**9.4±1.3**		**7.9±0.3**	**7.5±0.3**	**7.4±0.6**
**<N_31_/S_29_>**	**Sh_1_−St_2_/Sh_2_−St_2_**	**Sh_1_−St_3_/Sh_2_−St_3_**	**Sh_1_−St_4_/Sh_2_−St_4_**		**Sh_1_−St_2_/Sh_2_−St_2_**	**Sh_1_−St_3_/Sh_2_−St_3_**	**Sh_1_−St_4_/Sh_2_−St_4_**		**Sh_1_−St_2_/Sh_2_−St_2_**	**Sh_1_−St_3_/Sh_2_−St_3_**	**Sh_1_−St_4_/Sh_2_−St_4_**
Run1	5.8 (0.2)	5.7 (0.2)	5.9 (0.3)	**Run1**	7.0 (0.4)	7.1 (0.4)	7.4 (0.3)	**Run1**	6.9 (0.4)	6.7 (0.4)	6.7 (0.4)
Run2	5.7 (0.2)	6.1 (3.1)	6.9 (0.4)	**Run2**	7.2 (0.4)	7.0 (0.3)	7.0 (0.4)	**Run2**	6.6 (0.5)	6.1 (0.5)	6.2 (0.5)
Run3	5.6 (0.2)	5.7 (0.3)	6.0 (0.3)	**Run3**	7.6 (0.7)	9.0 (0.8)	9.3 (0.9)	**Run3**	6.9 (0.4)	7.0 (0.3)	7.1 (0.3)
Mean±SD	**5.7±0.1**	**5.8±0.6**	**6.3±0.2**		7.3**±0.5**	7.7**±1.1**	7.9**±1.2**		**6.8±0.5**	**6.6±0.5**	**6.7±0.3**
**<G_33_/L_27_>**	**Sh_1_−St_2_/Sh_2_−St_2_**	**Sh_1_−St_3_/Sh_2_−St_3_**	**Sh_1_−St_4_/Sh_2_−St_4_**	**G_33_/L_27_**	**Sh_1_−St_2_/Sh_2_−St_2_**	**Sh_1_−St_3_/Sh_2_−St_3_**	**Sh_1_−St_4_/Sh_2_−St_4_**	**G_33_/L_27_**	**Sh_1_−St_2_/Sh_2_−St_2_**	**Sh_1_−St_3_/Sh_2_−St_3_**	**Sh_1_−St_4_/Sh_2_−St_4_**
Run1	7.0 (0.3)	7.1 (0.4)	7.6 (0.4)	**Run1**	8.0 (0.7)	7.5 (0.3)	7.4 (0.3)	**Run1**	6.6 (0.5)	6.9 (0.4)	7.2 (0.3)
Run2	6.7 (0.2)	7.4 (0.3)	8.0 (0.6)	**Run2**	7.9 (0.7)	7.5 (0.4)	7.4 (0.3)	**Run2**	7.0 (0.5)	7.2 (5.0)	7.5 (0.4)
Run3	7.2 (0.4)	6.9 (0.4)	7.4 (0.4)	**Run3**	6.7 (0.6)	7.3 (0.4)	8.8 (1.0)	**Run3**	7.1 (0.3)	7.5 (0.3)	7.7 (0.3)
Mean±SD	**6.9±0.2**	**7.2±0.2**	**7.6±0.3**		**7.5±0.7**	**7.4±0.2**	**7.9±1.1**		**6.9±0.3**	**7.2±0.3**	**7.5±0.3**

Hydrophobic contact of C_α_−C_α_ distances (Å) between the residues L_27_/G_33_, S_29_/N_31_, N_31_/S_29_ and G_33_/L_27_ of human amylin and L_27_/G_33_, P_29_/N_31_, N_31_/P_29_ and G_33_/L_27_ of rat amylin and their hetero-assembly. *Sh = sheet and St = strand. Values are shown after excluding the first and the last chain of the each of the β-hairpin structures.

Monitoring the secondary structure contents during the simulation helps to understand the role of interactions that involve the β-strand motif on the stability of the aggregates [58]. Human amylin has at least two fragments that can form amyloid cross-β spines: the C-terminal region, which has a high propensity to form a zipper spine, and a H_18_ containing segment within the N-terminal region [59], [60]. For this reason, the average β-secondary structure of the human amylin aggregates is computed during the first and last 100 ns of the 300 ns trajectories using the DSSP [2] tool. In **Table 4**, the secondary structure content for the β_1_ region (residue 8–17) and β_2_ region (residue 28–37) are summarized. For all simulations of the human amylin the β-sheet secondary structure are stable over the duration of the simulations, with more than 80% of the residues in the N-terminal region retaining their β-sheet structure in the simulation of the single layer, compared to about 65% in the simulations of CC interface double layer models and about 50% in the simulation of the NN interface double layer. This is another indication that the β-strand−turn−β-strand topology is stable during the simulations, with about 8 residues from the N terminal (residues10–17) retaining more than 90% of β-sheet secondary structure, versus about 6 residues from the C terminal region consisting of residues 27–32.

**Table 4:** Average secondary structure content from the first (0−100 ns) and last (200−300 ns) 50 ns MD Simulations.

β_1_ segment, N terminal (residue 8–17)	Secondary structure, first 100 ns		Secondary structure, last 100 ns	
	β-sheet*	Turn*	β-sheet*	Turn*
Rat-SL	81.7 (6.0)	18.3 (6.0)	81.9 (4.0)	18.1 (3.0)
Human-SL	81.4 (5.0)	18.6 (5.0)	79.4 (3.0)	21.6 (3.0)
Rat-human-SL	84.6 (5.0)	15.4 (5.0)	83.6 (5.0)	16.4 (5.0)
Rat-DL, CC	78.2 (2.0)	21.8 (2.0)	77.1 (1.0)	22.9 (1.0)
Human-DL, CC	87.8 (2.0)	12.2 (2.0)	87.7 (1.0)	12.3 (1.0)
Rat-Human-DL, CC	77.0 (6.0)	23.0 (6.0)	77.6 (3.0)	22.4 (3.0)
Human-DL, NN	85.0 (3.0)	15.0 (3.0)	82.7 (1.0)	17.3 (1.0)
Rat-Human-DL, NN	87.7 (3.0)	12.3 (3.0)	86.0 (8.0)	14.00 (8.0)
**β_2_ segment, C terminal (residue 28–37)**	**β-sheet**	**Turn**	**β-sheet**	**Turn**
Rat-SL	45.3 (2.0)	54.7 (2.0)	42.6 (1.0)	57.4 (2.0)
Human-SL	66.7 (4.0)	32.7 (3.0)	65.2 (5.0)	34.8 (5.0)
Rat-human-SL	56.9 (4.0)	43.1 (4.0)	54.7 (4.0)	45.3 (3.0)
Rat-DL, CC	46.0 (8.0)	54.0 (9.0)	43.9 (1.1)	56.1 (10)
Human-DL, CC	69.2 (3.0)	29.8 (3.0)	66.9 (8.0)	33.1 (8.0)
Rat-Human-DL, CC	55.9 (2.0)	44.1 (1.0)	54.4 (2.0)	46.6 (11.0)
Human-DL, NN	61.0 (6.0)	39.0 (7.0)	49.4 (7.0)	50.6 (7.0)
Rat-Human-DL, NN	52.7 (1.0)	47.3 (2.0)	46.0 (10.0)	54.0 (10.0)

*Where: β-sheet  =  β-strand + β-bridge and Turn  =  turns + Coil. There is zero percent helix (α-helix+3^10^-helix+π-helix) secondary structure content.

Values are averages of three independent simulations over the entire simulation time and over all chains present in the oligomers.

**Figure 4:**
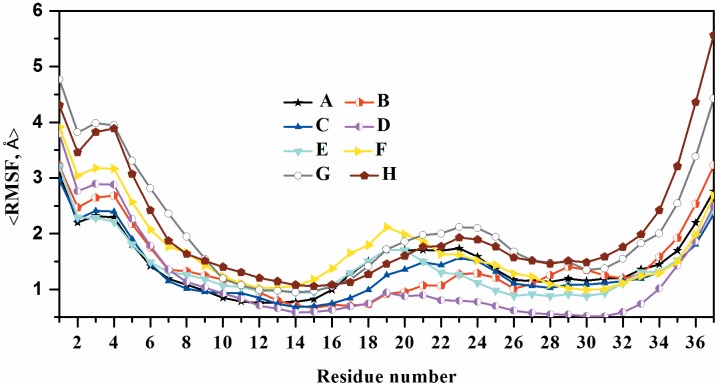
Root-mean-square fluctuation (RMSF) of the Cα atoms calculated from the three molecular dynamics simulations. A) Single layer conformation of human amylin, (B) single layer conformation of rat amylin, (C) single layer conformation of human-rat complex, (D) Double layer conformation of human amylin with CC interface (E) Double layer conformation of rat amylin with CC interface, (F) Double layer conformation of rat and human amylin mixtures with CC interface, (G) Double layer conformation of rat amylin with NN interface and (H) Double layer conformation of rat–human amylin complex with NN interface.

The root mean square fluctuations (RMSF) of the peptide backbone atoms, presented in **Figure 4**, reveal a similar pattern of stability and fluctuation for the human amylin models. As expected, the root mean square fluctuations signal a larger flexibility for the termini and the loop regions, suggesting higher plasticity of these regions, particularly in the C terminal. The higher flexibility of the β-strand regions of the NN interfaces double layer model compared to the ones in the CC interface suggests again the possibility of amyloid polymorphism that could result from the different interfacial interactions [61], [62].

### Rat Amylin

In order to explore how the differences in sequence between human and rat amylin in the structured C-terminal region (residues 21–37) changes the propensity to form amyloids, we have analyzed the structural stability of single layer and CC-interface coupled double layer aggregates of rat amylin. The average Cα root mean square deviation (the average taken over three independent trajectories) for the preformed rat amylin reaches 4.6 and 3.8 Å for the single and double layer respectively, which is only slightly larger than the values found for human amylin. However, this value is misleading. Overlaying the initial and final configurations reveals for the rat models a disturbance of the U-shaped fibril topology in the C-terminus and loop regions (**Figure 2** and **Figure S1**) that is missing for human amylin. The backbone dynamics (RMSF) of the rat amylin single layer and double layer with a CC terminal interface shows significantly higher flexibility in both the loop region and the C-terminal region than observed for the corresponding human amylin models, while the average backbone dynamics are found to be similar for both human and rat amylin double layers coupled by an NN interface (**see** **Figure 4**). Note that the double layer models for both human and rat amylin are less flexible than the single layer structures, which may result from the additional interaction due to the contacts between the two β-strands along the interface of the two layers that are absent in the single layer [20].

The differences in stability between amylin aggregates from the two species are due to three β-sheet breaking prolines in the segment 25–29 of rat amylin that are missing in human amylin. While the β-sheets secondary structure is largest in the N-terminal region (residues 8–17) of rat amylin with about 80% of residues in β-sheets, close to the value found for human amylin (**see** **Table 4**), rat amylin has an overall reduced β-sheet content of about 45% compared to the 65% found in the human amylin (**see** **Table 4**). This is because the middle residue in the _23_FGAIL_27_ sequence of the human amylin is responsible for the formation of an intermediate and transient β-sheet during fibril nucleation that forms before the formation of the N and C terminal β-sheets. The reduced β-sheet content in the C terminal region of the preformed rat amylin (due to the presence of the proline) could therefore slow the nucleation process, making rat amylin soluble and non-amyloidogenic under physiologic condition. Wu *et al* [63] have observed a similar difference in secondary structure of monomers. However, our simulation indicates also that while rat amylin is less stable than the human amylin, certain environmental conditions may cause it to form fibril structures. This has been observed experimentally for rat amylin dissolved in 20 mM Tris-HCl [64].

The turn secondary structure dominates the C terminal β-sheets region and is responsible for the lower intra-peptide hydrogen bond density in rat amylin when compared to human amylin. When proline residues as found in the the rat amylin sequence are substituted into human amylin the number of main chain and side chain hydrogen bonds is lowered by about 40 and 15 hydrogen bonds (**see** **Figure 3**), respectively. This is due to the smaller number of hydrogen bonds in the C terminal region and can be seen by comparing the inter-sheet distances between human and rat amylin for the residues 23 to 27, which is predicted to be the most amyloidogenic region of human amylin [65]. This quantity is calculated by averaging the mass center distance between each residue in one strand and its corresponding residue in the interacting strand of the adjacent sheet. A short distance between the two sheets indicates strong and favorable interactions while a larger distance is a signal for unfavorable contacts between the peptides. The average intermolecular distances between two β-sheets are smaller for the human amylin CC interface model than the same distances for the corresponding rat amylin model (**see** **Table 3**). This difference suggests again that in human amylin the C terminal regions serve as an anchor between the two hairpin units in the double layer, limiting its flexibility. On the other hand, the presence of the β-sheet breaking prolines in positions 25, 28 and 29 of the C terminal region of rat amylin increases their inter-sheet distance (**see** **Table 3**), making the growth of rat amylin aggregates through CC interfaces less favorable than for human amylin.

### Mixed Human-rat Amylin Aggregates

We next analyze the interaction of preformed mixed rat and human amylin aggregates in order to understand in more detail their cross-seeding. In agreement to a previous residue level amide vibrational coupling study on a rat-human amylin complex [18] we find that the single layer mixture of rat and human amylin is not stable. This suggests that in-register mixing leads to unfavorable interactions between the human and rat β-sheets. On the other hand, the U-shaped structure of the amylin conformation is preserved for both double-layer models (**see** **Figure 2** and **Figure S1**). However, the root-mean-square-deviation for the NN interface coupled models is ∼6.2 Å, larger than that of the CC interface (∼3.9 Å) coupled models. This difference in RMSD value is due to the shorter steric zipper of the NN interface. A similar trend can be seen for the inter-peptide hydrogen bonding of the two double layers, which increases during the simulation of the mixed aggregates coupled by CC interfaces slightly more than in the simulation of the NN-interface model. This relation is observed for both main chain and side chain hydrogen bonds. However, these differences in hydrogen bonding do not reflect larger stability of the mixed human-rat amylin model with CC-interface over that with NN-interface. Instead, they are due to the higher flexibility of the rat-amylin C-terminal residues in the NN-interface coupled model. This higher flexibility results from the three C-terminal prolines, which loosens the packing of the β-sheet residues. On the other hand, the N-termini are ordered in the mixed double layer model with NN interfaces, and the resulting contact between human amylin and rat amylin through the N-termini interface stabilizes the fibril core. Thus, the N-terminus is not only important for oligomer formation, but it also stabilizes the fibril via packing interactions resulting from burying the N-terminus within the fibril (**Figure 2** and **Figure 4**). Note that unlike the N-terminus, the C-terminus is much more mobile in the mixed aggregate than observed in both NN and CC double layer models of human amylin.

The average intermolecular distance between two β-sheets for human amylin CC interface models is smaller than found in both rat amylin and rat-human amylin complexes (**Table 3**). This suggests that the C-terminal regions of rat amylin in complex with human amylin is more flexible than in human amylin and therefore cannot serve as an anchor between the two hairpin units in the CC interface double layer. The presence of the β-sheet-breaking amino acid, proline, in positions 25, 28 and 29 of the C terminal region of rat amylin increases the inter-sheet distance making the growth of rat amylin on human amylin aggregate through CC interface less favorable. However, the inter-sheet distance in the NN-interface double layer models (**Table 2**) of both human amylin and the complex between rat amylin and human amylin are similar and in agreement with the experimental evidence. This result is also supported by our analysis of the average backbone dynamics, which revealed high fluctuation for the terminal residues indicative of local unfolding (**Figure 4**). In contrast to the CC-interface coupled double layer human –rat complex, the NN interface variant exhibits a much smaller difference in the root-mean-square-fluctuation values (**see** **Figure 4**). In addition, the β-sheet content at the interface between rat and human amylin is higher in our simulation than in the ones with CC interface. This suggests that the NN interface interactions are more important for the stabilization of the mixed rat-human amylin aggregates than the CC interface interactions, as amyloid formation between two different peptides is driven by sequence similarity and β-sheet secondary structure [34]. This result is in agreement with experiments [18] that have demonstrated the growths of the N-terminal region (a region in which both rat amylin and human amylin have identical sequences in the first 17 amino acid residues) of rat amylin on human amylin seeds.

Using the MMPBSA method we have calculated the free energies of protein-protein interactions. This allows us to evaluate in a quantitative way the thermodynamic stability of the various fibril arrangements (human amylin, rat amylin, and cross-seeded mixtures). While the MM/PBSA approach in general does not reproduces the absolute binding free energy values [19], [51], it was chosen because it allows for a rapid estimation of the variation in the free energy of binding, and because it usually exhibits a good correlation with experimental data [66]. In the present study we use single trajectory MM-PBSA [67] to estimate the binding free energy from an average of over 2000 equally spaced (at an interval of 20 ps) snapshots taken over a 40 ns production trajectory. Note that the solute entropic contributions (TΔS) can only be estimated crudely using normal mode analysis [19]. Our data are summarized in Table 5. Note the trend in thermodynamic stability: the NN interface stacking of rat amylin has lower binding energies (–49.2 kcal/mole) than the CC interface (–13.7 kcal/mole) while in the case of human amylin the CC interface construct has a more favorable binding energy (–70.2 kcal/mole) than the one with NN interface (–57.7 kcal/mole). Within the single layer structures, human amylin is more favorable (–45.7 kcal/mole) than both the rat amylin and mixed human-rat structures (–2.6 and −26.8 kcal/mole, respectively). Hence, the free energy differences support the trend observed earlier in our stability studies, which were derived from an analysis of various averaged structural quantities. In order to identify the dominant factors in the binding affinity we have analyzed further the various free energy components. We find that the polar solvation (**ΔE_PB_**), van der Waals (**ΔE_vdw_**) and non-polar solvation terms (**ΔE_non-polar_**) favor in all cases association. The nonpolar contribution adds favorably to the protein-protein binding while it is strongly opposed by electrostatic term.

**Table 5:** MM-PBSA free energy calculations and different Components of the binding free energy.

Structures	ΔE_vdw_	ΔE_elec_	ΔE_PB_	ΔE_SA_	ΔE_polar_	ΔE_nonpolar_	ΔG_binding_
**Rat-SL**	–184.1±1.7	2117.8±45.4	–2043.0±33.8	–106.7±1.7	74.8±11.6	–77.4±3.4	–2.6±2.8
**Human-SL**	–188.9±3.0	1042±93.5	–1007.0±91.6	–108.0±0.4	35.3±3.9	–80.9±5.6	–45.7±0.9
**Rat-Human-SL**	–185.6±3.6	1527.9±35.3	–1478.9±44.9	–109.8±0.5	49.0±9.5	–75.8±4.1	–26.8±8.7
**Rat-DL, CC**	–326.5±64.5	1774.9±39.3	–1665.0±54.6	–202.9±33.3	109.9±3.2	–123.6±31.1	–13.7±5.9
**Human-DL, CC**	–233.6±24.7	432.6±34.4	–393.6±33.8	–124.3±1.3	39.0±0.6	–109.3±15.3	–70.2±15.9
**Rat-Human-DL, CC**	–359.0±3.9	1119.3±4.6	–1032.0±5.2	–219.4±0.7	87.3±9.8	–139.6±4.6	–52.3±5.2
**Human-DL, NN**	–330.2±4.8	395.1±26.5	–318.7±30.9	–196.2±2.7	76.3±4.5	–134.1±2.1	–57.7±2.3
**Rat-Human-DL, NN**	–420.6±16.2	1259.0±49.3	–1143.0±51.5	–255.3±5.5	116.1±2.1	–165.3±10.7	–49.2±8.6

The data are averages of two independent 40 ns simulation with the corresponding standard deviations. All values are in kcal/mol. The polar term is the sum of Coulomb interaction energy (E_elec_) and polar contribution to the solvation free energy (E_PB_). The nonpolar term consists of takes the van der Waals interaction energies (E_vdW_) and the nonpolar contribution to the solvation free energy (E_SA_).

### Toxicity Mechanism

**Figure 5:**
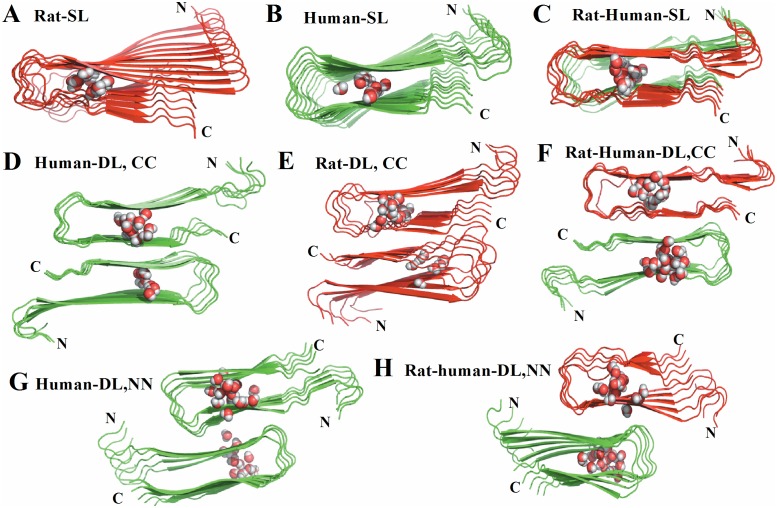
Representative snapshot of water molecules in the amylin single layer, double layer, and the complexes between human and rat amylin model. (A) Single layer conformation of human amylin, (B) single layer conformation of rat amylin, (C) single layer conformation of human-rat complex, (D) Double layer conformation of human amylin with CC interface (E) Double layer conformation of rat amylin with CC interface, (F) Double layer conformation of rat and human amylin mixtures with CC interface, (G) Double layer conformation of rat amylin with NN interface and (H) Double layer conformation of rat–human amylin complex with NN interface. Different colors mark rat (red) and human amylin (green).

A number of mechanisms have been proposed to explain the toxicity of rat and human amylin to cell cultures. Prominent examples are pore formation leading to membrane disruption or membrane destabilization by a detergent-like mechanism. Recent theoretical and experimental studies of human amylin aggregates in membranes indicate the same β-hairpin structure as has been observed in water [19], [68], [69], [70], [71], [61]. For this reason, we have monitored in the various aggregates the flow of water molecules into the hydrophilic water channel formed by their β-sandwich structure (see **Figure 5**). In all cases, pure human amylin, rat amylin, and mixed human-rat amylin aggregates, we find that water molecules enter the β-hairpin conformations. This suggests that membrane leakage due to pore formation could be the cause for the toxicity of rat amylin observed in recent cell culture toxicity studies [64]. The water molecules in human and rat amylin, and their hetero-assembly, are found in the interior of the oligomer cavity formed by a group of polar amino acids (N14, S28 (P28, in case of rat amylin), and T30) near the middle of the two β-strands. The location of the hydration channel in our simulation is similar to that found in previous simulation studies [19], [61]. Hence, our simulation indicates that direct protein–protein interactions between human and rat amylin in cross-seeded aggregates could enhance membrane leakage and cytotoxicity [72]. Ideally, one would want to compare simulations in both aqueous solution and bio-membrane environment. This would allow one to elucidate the mode of membrane interaction and pore formation, and the corresponding underlying conformational changes in the peptide. However due to limitation in the available computational resources we had to resort to monitoring the presence of water in the structure. However, our observation provide at least qualitative evidence for this potential mechanism of membrane destabilization that is in agreement with previous experiments and other simulations [73], [74], [75], [76].

## Conclusion

We have investigated in silico the stability of various rat and human amylin aggregates. The single layer mixture is not as stable as human amylin, pointing to unfavorable interactions in the in-register mixing of the human-rat amylin β-sheets. When associated through a N-terminal to N-terminal interface the double layer rat–human amylin complex maintains more residues in a well-defined β-sheet structure than in the case where the human and rat amylin molecules interact through a C-terminal-C-terminal interface, making this arrangement more preferable for the association of human amylin with rat amylin. Our result is in agreement with recent experiments that also found human and rat amylin associating through a NN interface, and it explains the observed weak amyloid-inhibiting properties of rat amylin [18]. Stabilization of the mixed human-rat amylin aggregates is sensitive to both hydrophobic and electrostatic interactions at the sheet-to-sheet interface. We have identified the L_13_ANFL_17_ motif of hydrophobic residues in the β_1_ region of amylin (which is the same in both rat and human amylin sequences) [51] as crucial for the stabilization of the cross-seeded aggregates. This insight might be useful for the design of aggregation inhibitors that improve upon the weak aggregation-inhibiting properties of rat amylin: computational screening of fiber-binding compounds could reveal small organic molecules or peptide-mimetics that stabilize the β-sheet regions reducing in this way amylin toxicity in type-2 diabetes. We also observe water penetrating the β-hairpin conformation of the two homo-oligomers and the hetero-oligomer, suggesting pore formation and membrane leakage as the likely cause for the toxicity of rat amylin observed in recent cell culture toxicity studies. If confirmed this would speak against the use of rat amylin as inhibitor of human amylin aggregation, since it has undesired cell toxicity, and, cross-seeded with human-amylin, forms aggregates. Green et al. [16] have shown that pramlintide, which is a three-proline substitution (with C terminal A25P, S28P and S29P mutation) analogue of human amylin, can still form fibrils, although less than human amylin but more than rat amylin. High concentrations of the pramlintide at pH 6–7.5 have a tendency for aggregation [77]. However a 10 residue peptide human amylin analogues with three proline residues at position 25, 28 and 29 (as in pramlintide) does not aggregate which can be attributed to the β–sheet disrupting effect of proline. A recent simulation also indicated that the three proline mutations (A25P, S28P and S29P) are important for eliminating human amylin aggregation [78]. Hence, while pramlintide was developed as a non-aggregating human amylin analogue, these finding indicate the possibility of formation of pramlintide fibrils, cross-seeded by circulating human amylin in diabetic patients that take this drug. We believe that our computational results demonstrate the need for further cell culture toxicity studies that investigate mixed human-rat amylin aggregates and such of human amylin and FDA approved drug pramlintide.

Finally, we remark that in our system the cross-seeded conformation assumes a different polymorphic form than the homo-oligomer: in the human-rat amylin complex we observe association along an NN-interface while for pure human amylin oligomers we observe association along a CC-interface. Hence, in amyloid diseases that involve transmission between species (such as in prion diseases), the toxic oligomers may differ in structure from that in the originating species. This has implications for the peptide-based drug design suggested in the previous paragraph, as in these cases the target for the inhibitor search should be the cross-seeded structures instead of the structures of the homo-oligomer. Since human-rat amylin aggregates allow one to study easily the role of sequence and conformation similarity in cross-seeding, we suggest to use these aggregates as model systems for amyloid aggregation induced by cross seeding of an amyloidogenic protein of one species administered into another species, or when simultaneous presence of more than one amyloid form is responsible for infection and toxicity [9], [79], [80], [81], [82].

## Supporting Information

Figure S1 The detailed structural changes for the three trajectories in each model, at the conclusion of 300 ns of molecular dynamics in explicit solvent. The initial structures are depicted in cyan. Different colors mark rat (red) and human amylin (green). Root-mean-square-deviation values calculated for each peptide with respect to the start configurations are included in parentheses.
(TIF)
