# Supplementary figures and images for: Inter-Species Cross-Seeding: Stability and Assembly of Rat - Human Amylin Aggregates
Source: PLoS One. 2014 May 8;9(5):e97051. doi: 10.1371/journal.pone.0097051 (PMC4014569; doi:10.1371/journal.pone.0097051)

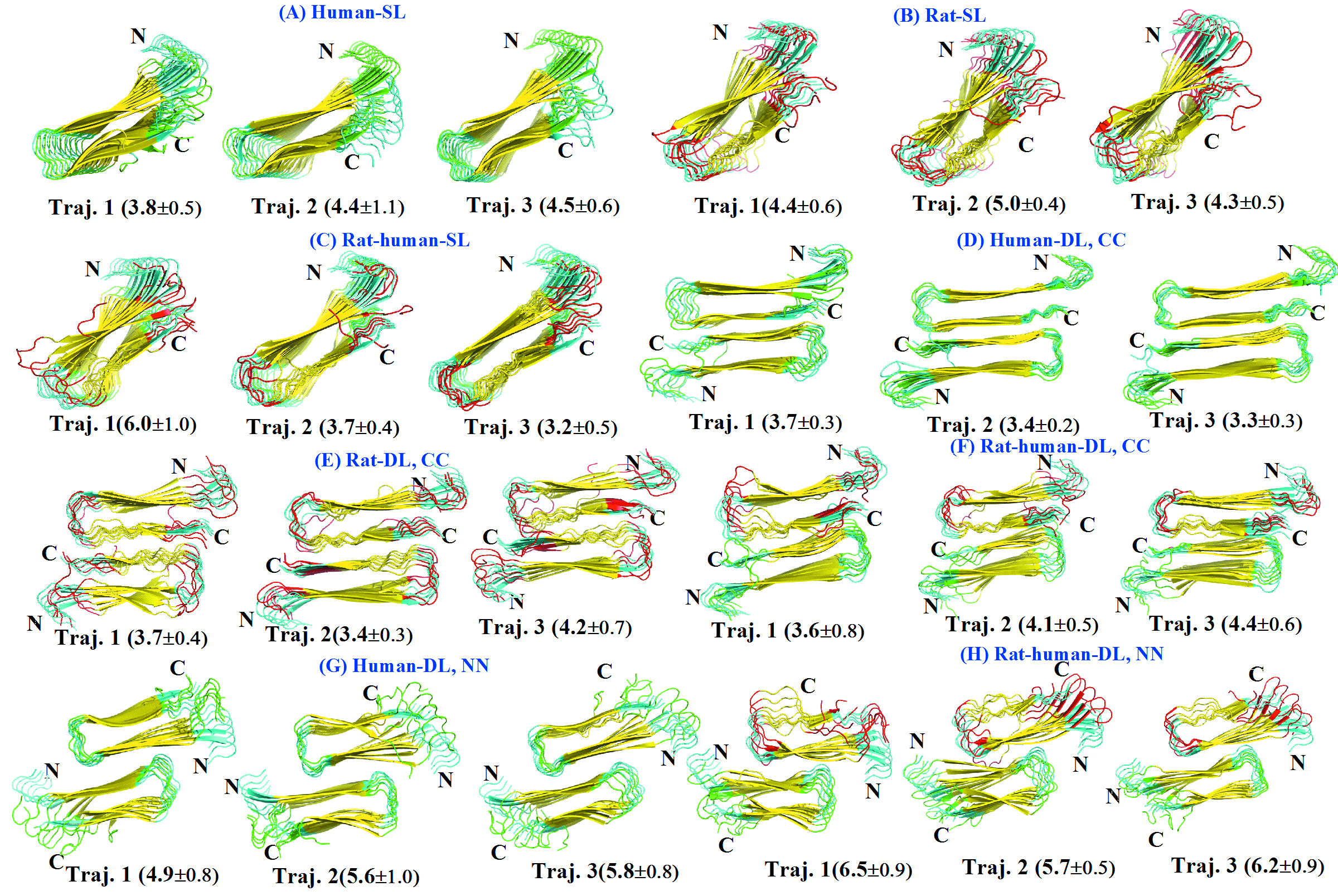

Supplement: Figure S1 — The detailed structural changes for the three trajectories in each model, at the conclusion of 300 ns of molecular dynamics in explicit solvent. The initial structures are depicted in cyan. Different colors mark rat (red) and human amylin (green). Root-mean-square-deviation values calculated for each peptide with respect to the start configurations are included in parentheses. (TIF) [file pone.0097051.s001.tif]
